# Deontological and Altruistic Guilt Feelings: A Dualistic Thesis

**DOI:** 10.3389/fpsyg.2021.651937

**Published:** 2021-06-22

**Authors:** Francesco Mancini, Amelia Gangemi

**Affiliations:** ^1^Scuola di Specializzazione in Psicoterapia Cognitiva (SPC), Rome, Italy; ^2^Department of Human Sciences, Guglielmo Marconi University, Rome, Italy; ^3^Department of Cognitive Science, University of Messina, Messina, Italy

**Keywords:** guilt emotion, deontological guilt, altruistic guilt, moral norms, disgust

## Abstract

In this paper we argue in favor of the existence of two different guilt feelings: altruistic guilt (AG) and deontological guilt (DG). AG arises from having harmed, through one's own action or omission, an innocent victim, while DG arises from the transgression of an internalized norm. In most daily experiences of guilt feelings both types are present, but we argue that they are not traceable to each other and that each can be present without the other. We show that the two guilt feelings can be distinguished with reference to behavioral, cognitive, and neurophysiological aspects. Moreover, we demonstrate that they are differently related to other processes and emotions. AG is connected with pain, empathy and ToM. DG is strongly related to disgust. We briefly illustrate some implications for moral psychology and clinical psychology.

## Introduction

In a review of literature about guilt feelings, Carnì et al. (2013) distinguish three main approaches: intrapsychic, interpersonal and integrated. The intrapsychic approach is well-expressed by Freud, who assumes that guilt is the manifestation of Superego intervention, which sanctions impulses, desires and actions that violate internalized moral norms. Hence, guilt would express an intrapsychic conflict. In line with Kugler and Jones (1992) “Guilt may be defined as the dysphoric feeling associated with the recognition that one has violated a personally relevant moral or social standard.” Fromm (1985) claimed that the fear of being guilty is the fear of having outraged an authority, even an unreal internalized one, and guilt is a kind of power that authority exerts over people. Individuals feeling guilty are particularly disposed to do everything they can to get authority's approval in order to alleviate their guilt (Carnì et al., 2013). “The psychoanalytic approach describes guilt and the psychological distress that characterizes it as not necessarily related to others. From this theoretical perspective, all of the actions of people who experience guilt are aimed at diminishing their own discomfort, regardless of whether there is any real damage repair. Thus, we can feel guilty and act to alleviate our guilt but not necessarily another's suffering.” (p. 6, Carnì et al., 2013). Accordingly, all guilt feelings would result in an intrapsychic conflict, even survivor guilt, where the moral norm transgression is not at all evident.

On the other hand, the interpersonal approach (Hoffman, 1982, 1998; Baumeister et al., 1994; Tangney and Dearing, 2002) shifts attention from people's internal states to the relational effects of their actions/omissions. This approach attributes feelings of guilt to the assumption of having harmed or failed to help another person, and it is crucially influenced by affective bonds and empathy which tie the “guilty” to the “victim” (Kubany and Watson, 2003). Individuals feel guilty not toward and internalized authority but toward another person, particularly if they are tied to the other by affective bonds or, at least, by a common belonging. From this perspective, “the function of guilt is to maintain in-group cohesion by inducing humans to carry out reparatory acts, help their neighbors, communicate their affection and be attentive to others' feelings.” (p. 6, Carnì et al., 2013). It is worth noting that the interpersonal approach also suggests a monistic conception of guilt feelings, even those in which it seems that the good of another person is not involved (Carnì et al., 2013).

Prinz and Nichols (2010) argued for an integrated approach that describes the psychological guilt-related state as follows, “Someone I am concerned about has been harmed and I have responsibility for that by virtue of what I have done or failed to do.” This schema includes two components: the perceived transgression of an internalized moral norm that defines responsibility, and the idea of not having preserved the other's well-being. According to the monistic thesis of Prinz and Nichols (2010), every type of guilt would thus result from these two, often mixed, ingredients: having transgressed an internalized moral norm and having inflicted harm to a victim.

In this article, we propose instead a dualistic thesis. There are guilt feelings tied only to having compromised, and not having achieved, altruistic goals, without there being any transgression of moral norms, and guilt feelings tied only to the transgression of an internalized moral norm even where there is no victim. Since most internalized norms regard safeguarding the good of others, the two senses of guilt are often co-present, albeit with different levels of intensity. For example, suppose a person finds on the street a wallet with money and documents, and that he takes it instead of trying to give it back to the owner. Afterwards, he may feel only altruistic guilt, thinking only about the difficulties and displeasure of the owner or only deontological guilt, over not having respected the internalized moral norm, “Thou shalt not steal,” or he may feel both, and in this case one or the other emotion may be prevalent.

## Altruistic Guilt (AG)

To feel AG it is necessary that an altruistic goal be compromised, that is, the goal of pursuing the good of another, with no personal advantage^1^. The main feature of altruistic goals is their content, the most obvious being the good of another person. A less obvious content, which, however, appears to be evident in the case of close affective bonds, such as the bond of care giving, is the desire for closeness with the other, especially if the other is in difficulty (Parisi, 1977)^2^. The desire for closeness can take very concrete form in the desire for physical closeness, or more abstract form, such as the desire for sharing or participation. If a dear friend of mine suffers a grave loss, such as the death of a parent, I am motivated to stand by him and to participate in his pain. I would feel guilty if I did not go to the funeral or if later that same evening I went dancing, that is, if I took on a state of mind very distant from that of my friend^3^.

It could be objected that being altruistic is a moral norm in itself and that, therefore, when one pursues the good of another, one does so in order to respect the duty to be altruistic. To respond to this objection a premise is necessary. The psychological significance of conduct depends on the goal pursued and not on the objective results. For a goal to be altruistic, it must be terminal, that is, not instrumental to another goal. This has an interesting implication: pursuing the good of another person with the ultimate goal of respecting a moral norm could not be an altruistic act. Thus, “*helping because you care is different, psychologically speaking, from helping because you think it is what morality demands*” (Prinz and Nichols, 2010, p. 113). One can pursue the good of others for at least two different motives. First, for an altruistic aim, that is, because one believes that the good of the other is important in and of itself. Second, out of a sense of duty, that is, guided by the goal of respecting an internalized moral norm, such as “be charitable.” Therefore, the statement “to be altruistic out of moral duty,” is an oxymoron. One cannot be altruistic out of moral duty but certainly one can *also* pursue the good of another out of moral duty. Between these two ways of pursuing the good of another, out of altruism or out of moral duty, there is a great difference psychologically. To gauge this difference, all one has to do is consider the emotional impact of discovering that our parents took care of us out of duty and not out of affection. The great difference between the two motivations is illustrated by a well-known novel, *Sophie's Choice* (Styron, 1979), which investigates the possibility of pursuing the good of others in order to fulfill the duties connected to one's own role and at the same time feeling guilty for having compromised one's own affective goal. Sophie is the mother of a son and a daughter. The three of them are interned in a Nazi concentration camp. One of the guards presents Sophie with the following dilemma: “both of your children are going to be killed, but I can save one of them on condition that you are the one who chooses which of the two is to die and which of the two is to be saved.” As a parent, Sophie has the responsibility to protect as best she can both of her children and so she has the duty to make a choice in the best overall interest. But, as a mother, Sophie has a strong affective bond with each of her two children, and the good of each of them is a terminal goal for her. Therefore, compromising the good of one of them cannot be an instrument for the good of the other. Sophie has the goal of fulfilling her duty as a mother responsible for her children by doing everything in her power to protect them. She chooses, therefore, to sacrifice one of her two children to save the other and she chooses to sacrifice her daughter because she figures that she has less chance of surviving than the son. With her choice Sophie fulfills her duty, but at the same time she is the victim of a perfectly understandable and terrible sense of guilt toward her daughter. She will choose to kill herself. In other words, Sophie's choice of sacrificing her daughter for the best overall good is acceptable from the point of view of her own responsibility as a parent, but it is unacceptable from the point of view of her affective bond with her daughter.

Finally, one last consideration with regard to altruistic goals: the more the good of the other is pursued at one's own expense the more the action is considered altruistic. To give to others that which for ourselves is superfluous, could be considered, altruistic, but not fully altruistic.

It is useful to distinguish those altruistic goals that concern the good of a specific person to whom we are tied by affective bond or friendship, which we might define as affective, from those that concern the good of others in general, with whom we recognize a common, albeit abstract, sense of belonging, such as belonging to the human race. In common usage, an affective bond is different from altruism and the latter term is limited to cases in which one pursues the good of the generalized other. In our view, the two subgroupings are similar in that the latter is plausibly a derivation of the former, by virtue of processes of abstraction and generalization, or as a result of complex evolutionary shifts (Tomasello, 2016).

To have AG feelings it is necessary to recognize that one's own altruistic goal has been compromised because of one's own act/omission, and that this was not inevitable, that is, that one was free of constraints (Castelfranchi, 1994). The intensity of AG feelings depends on numerous factors, among which the most interesting, for our purposes, is the closeness of the affective tie between the “guilty party” and the “victim” and also the degree of empathy elicited by the victim (for a review, see Carnì et al., 2013). The internal feeling is one of pain and anguish for the victim; the internal dialogue is typically something like this: “poor friend, how I've made you suffer!” (Basile and Mancini, 2011).

The following is an example in which a “guilty party,” causes, by omission, harm to a person in a visible state of vulnerability^4^.

*You were in your school days, when every chance not to go to school and instead to wander around town was an opportunity. That March morning, a bright sun was shining, it was almost too warm. You and your friends decide not to go to school and to go to the royal gardens. You spend a lovely morning with your friends but while you're on your way back home you hear some shouting coming from your right. Uneasy and scared, you go to see what it is and find yourself looking at this scene: a circle of a dozen or so boys is standing around a black boy lying on the ground. He is one of those street vendors who earn a living by going around the royal gardens selling all kinds of things. They are beating him bloody and saying horrible things to him. Petrified, you and your friends stand there looking at the terrible scene, impotent, without moving a finger. You go back home and you can't manage to get that scene out of your head. You feel a knot in your throat and you imagine all of the things that you could have done to stop them, to try to limit the harm done …. But you hadn't done anything*.

Here we give two examples in which an altruistic goal is compromised by the lack of closeness to the victim:

*I was suffering from severe symptoms and was admitted to hospital. During this time I shared a room with another person and we became friends. After 10 days, the doctor informed me that all was well and that I could go home. I was packing my bag when my friend came into the room. He was very distressed: the doctor had diagnosed him with cancer. Even today, I can't bear the thought that I was able to resume my life and his became an ordeal. I feel guilty at not having shared his fate*. (Castelfranchi, 1994).

In this example, the lack of closeness is due to the variance between one's own favorable fate and that of the unfortunate companion, “*I was about to resume my life, while his was about to become an ordeal*.”

The second example is the account of a psychiatrist. It is important to know that the doctor was very close to her grandmother, who had taken care of her during her childhood:

*I was on duty in my hospital ward, when I was called to another ward, where my grandmother had been admitted. As soon as I arrived, I realized that my grandmother was in a coma and dying. I decided to return to my own ward to advise a patient personally that I could not talk to him that day. I went back to my grandmother and saw that she had died in the meantime. Several days later, I still feel very guilty about not staying with her and not holding her hand while she passed away*.

In both examples, the guilt feeling derives from the impairment of the altruistic goal of being close to the victim by participating in his suffering. It could be asked why both the protagonists did not simply feel disappointed or pained rather than guilty. As we are about to see, altruistic guilt is the emotion one feels if one does not put into action the altruistic disposition activated by pain. That disposition is actualized not only by doing the good of the victim but also by standing by him.

Disposition to act in AG seems to be directed in at least three directions. First, it is plausibly directed at avoiding more actions/omissions that could cause harm to the victim. Second, it is oriented toward the good of the victim, placing it before one's own. Third, there might be an effort to achieve physical or even psychological closeness, and therefore participation, which can also take the form of reducing one's own good fortune. It is known, for example, that some people, even without realizing it, systematically block their efforts to reach a goal because, if they were to succeed, they would increase the distance between themselves and, say, a less fortunate brother (Weiss, 1993). The so-called Dobby Effect (Weiss and Sampson, 1986; Nelissen and Zeelenberg, 2009), i.e., avoiding success and inflicting self-punishment, which is activated when compensatory strategies are not practicable, *may not* be aimed, therefore, at expiation or at re-establishing equity (Castelfranchi, 1994; Mancini, 1997). Rather, it may be motivated by the desire not to distance one's own state from that of the victim.

There are no known specific facial expressions for guilt feelings, of for AG. Verbal expressions of guilt, internal and external, refer to the victim, his pain, his fate, and also to one's own acts and omissions. For example, a study conducted by Basile and Mancini (2011) showed that AG could be activated by phrases such as “*How could I have hurt her so badly?”, “She is hurting so badly and I haven't done a thing to help her!”, “I left him all alone at a difficult time,” “I could have given her a hand and instead I didn't lift a finger.”*

Moreover, AG feelings may include a sense of anguished pain for the victim, a propensity to saying “*I'm sorry” instead of “Excuse me,” “Pardon me,”* a compassionate attitude, the tendency to focus attention on the suffering victim, and finally, the attempt to alleviate the victim's suffering at one's own expense (Basile and Mancini, 2011).

### Do Altruistic Goals Exist?

This implies a fundamental theoretical question. Are there *a priori* reasons why the existence of truly altruistic goals would be impossible? That is, that the good of another is necessarily pursued for personal gain?

It could be sustained that one who pursues another's good pursues his own goal and therefore, in the final analysis, pursues his own good. A response to this objection is offered by the observation of Castelfranchi (2011) that there is a clear difference between *own* goals and goals *for oneself*. It even appears ridiculous, in the author's view, to confuse the idea that a *goal directed/governed* system, which is, by definition, regulated by its *own* goals, that is, internal and self-regulating (it could not, after all, be otherwise) with the idea that such a system is *egotistic*. That an agent is moved by his own internal goals and not guided from outside says nothing, in fact, about his being “altruistic” or “egotistic.” The difference thus depends on the content of the goals that move him, and not by the fact that he is moved by internal goals.

A second objection to the possible existence of altruistic goals is that achieving an altruistic goal involves positive consequences for the agent that do not regard the well-being of others but, for example, a sense of satisfaction, increased self-esteem, etc. Here, too, it is necessary to clarify a misunderstanding already resolved by Seneca (Castelfranchi, 2011):

*But even you—you'll say to me only cultivate virtue because you hope to obtain some pleasure from it. Well, just to begin with, the fact that virtue gives pleasure doesn't mean that one seeks it for that reason; pleasure is only an adjunct, not the goal of our efforts: we will achieve it but by aiming at another end, which is in fact virtue. Just, as in a field of grain, little flowers spring up here and there—but it is not to these blossoms, as much as they bring pleasure to the eye, that our labors are aimed, the sower's aim was another, the flowers are an extra—in the same way pleasure is neither the reward nor the cause of virtue, but an accessory to it; the virtuous do not practice it because it procures them delight, but if it should procure them delight, they take pleasure in it*. (Seneca, *De vita beata, IX*).

Finally, one might object that pursuing altruistic goals yields advantageous returns in any case, for example, in terms of reciprocity, greater cohesion of one's group of belonging, or greater likelihood of survival of one's own genes. These objective effects may also increase the probability that altruistic individuals will be selected, but this does not mean that one who pursues an altruistic goal is motivated by the anticipation of the future benefits for oneself, for one's group, or for one's own genes. By analogy, it is entirely plausible that evolution has rewarded individuals endowed with strong sexual desire, but it is not plausible that the provision of a vast and enduring progeny is the principle motivation for sexual conduct. A much more likely motivation is erotic desire.

In conclusion, there is no reason to deny *a priori* the existence of genuine altruistic goals. But is there evidence that, in fact, people can pursue the good of another even if they realize that there will no advantage in it for themselves? In other words, is there any evidence in support of the actual existence of altruistic goals or, on the contrary, are the goals that appear to be such actually egoistic? Do we in fact pursue the good of others only for our own gain?

The answer is found in the long series of refined experiments by Batson (Stich et al., 2010) and in other studies summarized by Warneken and Tomasello (2009). It seems that altruistic goals do indeed exist. Human beings appear to be capable of pursuing another person's good without any kind of personal return. People help others for a truly altruistic goal and not for any of the following forms of personal gain: to reduce the *distress* provoked in them by seeing another person in difficulty; to avoid being punished if they do not help; to avoid the distress related to the guilt feelings they would have if they didn't help; to obtain the expected gratification that could come from others or from oneself in the form of positive sensations, such as a feeling of pride for having helped another; to feel resonating inside themselves the relief of the person they have helped get out of a painful situation. Instead, individuals pursue the good of another even if they know they will have no information regarding the effect their actions may have on those they have helped (Stich et al., 2010).

Furthermore, the studies conducted by Tomasello and colleagues (Warneken and Tomasello, 2009; Tomasello, 2016) show altruistic behavior in very young children, around 2 years old. The authors suggest that altruistic disposition is innate. In support of the innate rather than cultural origins of altruism, some studies demonstrate that monkeys and rats let themselves die of hunger if they connect eating with causing pain to another individual (Bekoff and Pierce, 2010). It seems, therefore, that there do indeed exist altruistic goals in the strict or terminal sense, pursued at personal cost and plausibly innate.

### Pain

Pain is the emotion one feels when one assumes that another person has suffered undeserved harm (Castelfranchi, 2005). The pain activates a willingness to help or be close to the victim. One feels AG, on the other hand, when one realizes that the other did not deserve to suffer but that *you did not help him and you did not even stand by him*, despite knowing that you could have helped him or consoled him. But could the two protagonists (of the stories reported above), have acted differently than they did? What else could the doctor have done? Before responding to these questions, an observation is necessary. As Miceli and Castelfranchi write: “after an event, people tend to overestimate their preexisting predictive capabilities—showing the well-known hindsight bias (e.g., Fischhoff, 2003)—as well as their control over the situation. Therefore, they may start into a chain of counterfactual thoughts about what they could and should have (not) done (for instance, “*I scrambled over others to escape*; or *I thought only of myself, without trying to save others*”) (Miceli and Castelfranchi, 2018, p. 721). Therefore, after the event, the doctor in our example, very probably overestimated her chances of foreseeing that her grandmother would die in just a few minutes and that she could have waited. Thus, she had been wrong to leave and consequently felt a sense of guilt rather than pain or disappointment.

What about the patient who abandons his comrade in illness? What else could he have done? He could have remained there with him but at the cost of giving up on resuming his own life, if not completely, at least in important ways. Even though he chose to resume his own life, it is hard for him to eliminate the doubt that he would have done better to remain with him, especially given his vivid memory of his companion's pained and anguished face. As long as the doubt remains dormant, his dominant feeling is pain, but when the doubt reawakens, pain is transformed into a feeling of guilt.

## Deontological Guilt

DG, unlike AG, derives from the assumption of having violated one's own moral norms that one had the goal to respect, even if no harm comes from the transgression, not even harm to oneself. For a goal to be moral, it must be terminal, that is, not instrumental to other kind of goals or to personal advantages. An example:

*Julie and Mark are brother and sister. They are traveling together in France on summer vacation from college. One night they are staying alone in a cabin near the beach. They decide that it would be interesting and fun if they tried making love. At the very least, it would be a new experience for each of them. Julie was already taking birth control pills, but Mark uses a condom too, just to be safe. They both enjoy making love, but they decide not to do it again. They keep that night as a special secret, which makes them feel even closer to each other*. (Haidt et al., 2000).

This episode has been presented to thousands of people all over the world, belonging to different cultures and religions, and the vast majority of people have judged the incest of Mark and Julie to be morally wrong. The story stipulates that the two are consenting adults, takes for granted that they are capable of understanding and intent, and highlights that there is no risk of procreation, or psychological or social harm. Despite all these elements, however, the intuition that their contact is sinful is widespread. We can suppose that most people, if they were to find themselves in the position of Julie and Mark, would feel guilt.

Another example:

*I had just graduated in medicine. One evening, when I arrived for night duty, I found that a patient with terminal cancer had gone into a coma. Even in the torpor of his coma the patient complained of the pain. The head physician instructed me to give him massive doses of morphine, which would have soothed his pain but above all, would have speeded up his death. I was just going to inject the morphine when the thought crossed my mind “who am I to decide on this person's life or death? Who authorizes me to play God? It is not morally right. I cannot do that. This thought stopped me from acting”*.

In this example, the moral constraint that stopped the young doctor's act of euthanasia was the fear of committing a deontological wrong. According to our thesis, if the young doctor had injected the morphine, he would have felt deontological guilt.

A third example comes from the Bible and is, probably, the best-known deontological sin. The original sin was an act of pure disobedience to God, of *hubris*, of pride, an attempt to take God's place by claiming to know better than He what is Good and what is Evil. Eating the apple was not sinful because it harmed someone, but because of its lack of respect for God. Thus, the existence of guilt feelings independent of the well-being of others but tied to the awareness of having violated an internalized moral norm seems plausible. Another example is the moral condemnation of cloning, even for curative rather reproductive ends, because it violates the principle Do Not Tamper with Nature (Sunstein, 2005).

Feeling a sense of DG requires recognizing that one's own deontological goal has been compromised by one's own act/omission, and that this was avoidable, that is, that one was free of material and moral constraints. It is also possible to feel guilty only for intentions, desires, or dispositions, such as, for example, having pedophiliac desires, even if one does not act on them, if one assumes that “wrongdoing can be … a possible consequence of personal traits and dispositions—provided the person views such traits as modifiable through effort (thereby feeling responsible for not trying to modify them)” (p. 725, Miceli and Castelfranchi, 2018). If, on the contrary, it is assumed that one does not have the power to change one's own perverse disposition, then that disposition can be experienced as a misfortune rather than as a fault.

The dispositions to act in DG are to confess, to ask to be excused or pardoned, therefore to recognize that one has transgressed a moral norm that ought to have been respected and to signal that one has recovered the will to obey. As well as to prevent other possible failings. This means that the “guilty” will tend to judge their own acts more severely. They will even resort to washing rituals (Zhong and Liljenquist, 2006; Lee and Schwarz, 2011). It is not a coincidence that in all religions, in order to clean the conscience of sin, sinners make use of body washing rituals. Baptism, for example, is a washing ritual whose end is purification from original sin. In two studies (D'Olimpio and Mancini, 2014; Ottaviani et al., 2018a) it has been found that the induction of DG, but not AG, involves a greater tendency to washing and that washing improves the emotional state.

### Moral Norms

DG derives from the assumption of having violated an internalized moral norm. A moral norm is internalized if, for that individual, respecting the norm is a terminal goal, that is, if pursuing it is an end in itself and not instrumental to other goals, such as, for example, safeguarding one's own reputation.

What are the characteristics of moral norms and how are they distinguished from other norms, such as conventional norms? Moral norms are deontic and, as such, they limit the freedom to choose among possible choices. Traditionally, moral norms are deemed to be different from conventional norms for three reasons traceable to the so-called *Moral Signature* (cf. Haidt and Joseph, 2004). First, moral norms are ***considered*** universal, because they are ***considered*** valid for everyone, even if one is aware that not everyone shares them. For example, if I believe that pedophilia is morally wrong, I am inclined to think that all human beings, independently of their culture of belonging, their age or other individual characteristics should respect that norm. The information that pedophilia is or has been accepted in some cultures does not imply that those who believe it is morally inadmissible stop believing that no one must commit pedophiliac acts.

Second, moral norms are also considered unmodifiable, because it is not possible to change them by way of an agreement. For example, if I believe that pedophilia is morally wrong, I would continue to judge it as immoral even if there were a law that condoned it. Moreover, the violation of moral norms is habitually thought to be more serious. Third, moral norms concern goodness and respect for others. Conventional norms, on the contrary, in accordance with the *Moral Signature*, are contingent. They are dependent on the socio-cultural environment. For example, in our culture, burping at the dinner table is deemed improper but it seems that in some cultures it is a satisfactory way of showing appreciation for the meal. Conventional norms are modifiable. For example, the rules of tennis can be modified by agreement of the members of a tennis club. The violation of conventional norms is not generally considered particularly serious. For example, burping at the dinner table may elicit criticism or hilarity but not a moral condemnation. Finally, conventional norms do not concern the well-being of others. For example, the norm “you shouldn't put your elbows on the dinner table while eating,” defends the space of your table companions but to say it defends their well-being appears to be an exaggeration.

#### What Research Says About the Moral Signature

Are moral norms really considered and thus perceived to be universal? Some studies appear to cast doubt on the idea that internalized moral norms are *perceived* as universal. For example, Kelly et al. (2007) have demonstrated that people judge morally guilty an officer of an oil tanker who decides to inflict harsh corporal punishment on a sailor who fell asleep while on watch. That same officer, however, would not be judged guilty, or would be held less guilty, if instead of being aboard a present day oil tanker, he was on a sailing ship in the 1600s, where instead it was customary to inflict such punishment. This finding, however, does not necessarily cast doubt on the universality of moral norms. Indeed, it is useful to distinguish two types of judgment. It is one thing to say that in the 1600s it was right to administer harsh corporal punishment, while in our own time it is wrong. This would imply that moral norms can be relativized. It is another thing to say that the officer in the 1600s was not guilty because he was convinced, in total good faith, of the correctness of his actions, which were consistent with the morality of the age. This, however, does not imply that the morality of the age was “fair.” It is possible that the responses of the participants in Kelly's study were of this type.

Are moral norms really considered unmodifiable and conventional norms modifiable? It is probably better to change the question. Who is authorized to modify the norms and under what conditions? The aptness of this question is suggested by the comparison of two sets of findings. The study by Kelly et al. (2007) indicates that the officer of the aforementioned oil tanker would be judged not guilty, or less guilty, if he had inflicted the corporal punishment by order of the ship's commander. The famous studies by Milgram (1974) confirm that a recognized authority, personified by a scientist, can render morally permissible conduct that is harmful to others. This seems to suggest that moral norms are considered modifiable by a human authority and that, therefore, such norms are not perceived as being on the same level as natural laws. At least in this respect they would appear to be indistinguishable from conventional norms.

Conversely, other findings suggest that moral norms cannot be modified by a human authority. The studies by Turiel and Nucci (1978) and Nichols (2004), for example, demonstrate that children do not recognize the authority, for example of their teacher, to abolish the moral norm “do not pull the hair of your classmates,” while they do recognize her authority to abolish the rule of good manners, “do not chew gum in class.” The contrast in these findings can probably be resolved if we take into account the stature of the authority and the moral importance of the norm. The moral importance of a norm seems to depend on how much the transgression of the norm in question compromises what appears to be the natural order. The stature of the authority is related to how much the authority's directive appears to be an expression of the natural order, the course of history, a supreme good, or the will of God. The Pope can modify the norm not to kill and command the crusaders to exterminate the infidels if it is assumed that he is speaking on behalf of God. The teacher, on the other hand, is not attributed the authority to change the moral norm “do not pull the hair of your classmates” but only the rule of manners “do not chew gum in class.” Therefore, the difference in modifiability of moral and conventional norms appears to be tied to the stature of the authority that changes the norm and to the moral importance of the norm.

Does the content of internalized moral norms only concern the good of others? In effect, many internalized moral norms concern the good and the rights of others, for example, do not kill, do not rob, do not inflict useless pain. Others, however, though they, too, may be internalized, do not concern relationships with others [see Moral Foundation Theory^5^, Haidt and Joseph, 2004; Graham and Haidt, 2012]. Some religious norms^6^, for example, such as the first three of the ten commandments, regulate explicitly and exclusively the relationship with the divinity and not relationships with other people: 1. I am the Lord thy God and thou shalt not have other gods before me; 2. Thou shalt not speak the name of the Lord thy God in vain; 3. Thou shalt remember the Sabbath Day and keep it holy. There are also internalized moral norms which prohibit sexual conduct that do not involve harm to or violation of others, for example, coupling with animals, incest between consenting adults even with no risk of procreation, and masturbation^7^.

## Empirical Evidence of the Difference Between Altruistic and Deontological Guilt, and More Generally Between the Two Moralities

Various studies have demonstrated that these two senses of guilt are distinct from both a behavioral and neurological point of view. An initial series of studies on moral choices was conducted by using the “trolley dilemma” (Foot, 1967). In its original form, the trolley dilemma asks participants to imagine that a trolley car is careening out of control on a track with five persons on it who, if the trolley continues on its course, will be run over and killed. Participants are then asked if they would pull an exchange lever, sending the trolley down another track where, however, there is another person who will certainly be hit and killed. This dilemma is especially interesting for the distinction between the two types of guilt. Indeed, it requires participants to choose between two incompatible options, which in light of what has been said so far can be defined as altruistic/humanitarian vs. deontological. The altruistic/humanitarian option consists in moving the lever to cause the death of one person in order to save five, thus reducing as much as possible the overall suffering and harm. Nevertheless, moving the lever amounts to assuming responsibility for changing the course of events decided by fate, or for believers, by God. The deontological option consists in omitting to move the lever and allowing the five people to die, but not taking the responsibility to change the natural course of events and thus respecting the deontological principle *Do Not Play God*^8^. According to Sunstein (2005), this principle is capable of explaining why, all things being equal, omission tends to be considered less grave than action. In line with these studies, Gangemi and Mancini (2013) have shown that the people who choose not to act report that their choice was preceded by an internal dialogue coherent with the *Do Not Play God/Not tamper with Nature* principle, for example, “Who am I to decide who lives and who dies?”, while those who choose to act, appeal to the minimization of others' pain and suffering, and thus to an altruistic/humanitarian principle, for example, “Better that only one person die rather than five.”

Furthermore, the inducement of DG implies a preference for not moving the lever, while the inducement of AG implies moving it (Mancini and Gangemi, 2015). A study by D'Olimpio and Mancini (2016) confirmed this finding, adding the evidence that a preference for omissive choices is ascribable to DG and not to shame (for an explanation of the difference between guilt and shame, see the conclusion below). Another study highlighted that the deontological choice is more frequent if participants are asked to imagine being next to the exchange and to have nearby a figure who represents a moral authority, such as a judge or a police officer. The contrary happens when participants are asked to imagine themselves next to the lever and therefore close to the five people. The first situation plausibly induces respect for authority and therefore for the *Do Not Play God* principle, while the second activates empathy and, in turn, an altruistic/humanitarian impulse (Gangemi and Mancini, 2013). Similarly, Migliore et al. (2019) observed “a higher number of utilitarian/positive responses when individuals had to respond to an Empathic Moral condition (the decision has made while physically close to the potential victims), compared to a Deontological Moral condition (the decision was made while flanked by an ‘authority’).”

Other researchers have found that respect for the moral norm “Do not tell lies” can even restrain the telling of white lies that would create a benefit for the deceived and a small harm for the deceiver, and Pareto lies, in which both the deceived and the deceiver would benefit. Respect for the moral norm, therefore, seems to be able to win out over the effect of the altruistic and cooperative disposition (Biziou-van-Pol et al., 2015).

Some findings seem to suggest that DG, but not AG, reduces the moral authority that one recognizes in oneself. For example, the inducement of DG reduces refusals of unjust offers in the three-person *Ultimatum Game*^9^, unlike what happens with the inducement of AG (Mancini and Mancini, 2015). This suggests that inducement of DG lessens the feeling of being entitled to make justice prevail.

Basile and Mancini (2011) activated the two senses of guilt separately by using as stimuli facial expressions of basic emotions (i.e., Ekman photos) accompanied by internal dialogue phrases typically associated with the two types of guilt feelings. For example, for DG, angry and contemptuous faces together with phrases such as “How could I have done that!” while for AG, sad faces with phrases such as “How could I have left her alone.” Moreover, in a study using functional magnetic resonance (fMRI) to identify the neural substrate of the two guilt feelings, Basile et al. (2011) found activation of the insula and the anterior cingulate cortex in the condition of DG and activation of medial prefrontal areas in the condition of AG. These findings appear to be particularly interesting, not only because they demonstrate that the two guilt feelings can be “traced” to different cerebral circuits, but also for the specific areas involved.

Previous studies (cit. in Basile et al., 2011) related mPFC with “mind reading” tasks, which typically entail experiencing social and interpersonal emotions (Blair, 1995; Shallice, 2001). Moll et al. (2007) found that the medial prefrontal cortex seemed to be involved in empathic guilt and compassion feelings. Further, activation in the medial prefrontal cortex was observed in subjects experiencing moral sentiments, and also when viewing other people's sad faces (for reviews, see Moll et al., 2005a, and Moll et al., 2005b). Basile et al. (2011) conclude that: “This suggests that altruistic guilt, falling within compassion emotional domain, might share with these emotions a partially common neural substrate.” For the purposes of this article, it is interesting to observe that the DG substrate does not seem to include mPFC. This may suggest that the relationship that exists between AG and experience of empathy and compassion does not exist between these same functions and DG. Any conclusion on this point, however, must be made with caution since the medial prefrontal cortex is probably involved in many other cognitive and affective processes, and different parcels of mPFC structure could be embedded in different networks. On the other hand, the insula, which is activated by DG, is associated with the experience of disgust and self-blame (Rozin et al., 2000). The close proximity between deontological guilt and disgust, but not between altruistic guilt and disgust, is suggested by two further studies that used tDCS (transcranial direct current stimulation; Ottaviani et al., 2018b; Salvo et al., in press). The stimulation of the insula cortex activates the parasympathetic nervous system in a way totally compatible with the activation of disgust, increases disposition to words related to cleaning, and induces the tendency to feel disgust. Above all, the subject judges transgressions of moral norms to be more serious when the insula cortex is activated compared to when it is not, as in the following example: “you see a politician use tax revenues to construct an addition to his own home.” Whereas, activation of the insula cortex does not induce changes in altruistic judgments, such as “you see a boy set up a series of traps to kill stray cats in his neighborhood.” This finding was replicated by Salvo et al. (in press). They also found that indirect inhibition of the insula via cathodal transcranial direct current stimulation (tDCS) reduces disgust and moral rigidity.

This finding evokes the hypothesis that there is a relationship between disgust and DG feelings, which is worth examining in depth.

## The Relationship Between DG Feeling and Disgust

A question much debated in the last 20 years regards the relationship between moral transgressions and disgust. Is there such a thing as true moral disgust or is it only a linguistic artifact, that is, the connotation of a moral transgression as “disgusting” is simply a manner of speaking or is it the expression of a real reaction of disgust in the face of a moral transgression?

For example, Ottaviani et al. (2013) enhanced activity of the parasympathetic nervous system without concurrent changes in heart rate (HR) for physical disgust and decreased vagal tone and increased HR and autonomic imbalance for moral disgust. Results suggest that immorality relies on the same biological root of physical disgust only in subjects with obsessive compulsive tendencies.

On the contrary, Chapman and Anderson (2013), after examining studies that manipulate morality and measure disgust, conclude: “…participants who are exposed to moral transgressions show signs of disgust in many modalities, from self-report, to facial expression, to overt behavior and implicit priming. Moral disgust does not seem to be restricted to transgressions that reference physical disgust and also cannot be easily explained away as metaphorical communication.”

However, they do add one caveat: “many of the individual studies that point to this conclusion have limitations, and the literature is not without conflicting results that will have to be reconciled.”

The relationship between disgust and morality has also been studied from a different prospective, namely, whether the activation of disgust and physical cleansing influence moral judgments. Chapman and Anderson (2013) conclude: “…almost all of the studies that have manipulated disgust or cleanliness have reported effects on moral judgment. These findings strengthen the case for a causal relationship between disgust and moral judgment, by showing that experimentally evoked disgust—or cleanliness, its opposite—can influence moral cognition.”

Landy and Goodwin (2015), to the contrary, conclude: “On the basis of the results of this meta-analysis, we argue against strong claims about the causal role of affect in moral judgment and suggest a need for new, more rigorous research on this topic”.

In conclusion, so far it is still not entirely clear whether moral transgressions can elicit true disgust or if moral disgust is substantially a linguistic artifact. Nor is it entirely clear if, as the sentimentalist tradition would have it, disgust can influence moral judgments. The difficulties in drawing univocal conclusions could perhaps be overcome by making some distinctions, some of which are suggested by Tobia (2014) “between manipulations inducing feelings of self-cleanliness and self-disgust and manipulations inducing a sense of other-cleanliness and other-disgust. ……and whether the person making the moral judgment is described as the actor in the scenario or as an observer, judging someone else's action.”

In other words, the relationships between morality and disgust may be different depending on the distinctions that are taken into consideration. In keeping with the purpose of this article, we are interested in addressing a very specific question, namely, whether there is a privileged relationship between DG and disgust but not between AG and disgust, as suggested by the findings of Basile et al. (2011), Ottaviani et al. (2018a), Salvo et al. (in press), and also by the findings of Robinson et al. (2019): “In all three studies, we found that trait disgust sensitivity predicted more extreme deontological judgment” (Robinson et al., 2019).

As mentioned above, based on the observation that, in all religions, sin soils the soul and washing purifies it, the scientific literature has suggested the existence of a strong relationship between guilt and disgust (Lee and Schwarz, 2011). Various studies have confirmed the relation between the physical component of disgust, the contamination of moral evil, and the necessity to wash (cf. Doron et al., 2012). In this regard, Zhong and Liljenquist (2006) have described the “Lady Macbeth” effect, in which “a threat to moral purity implies the need to wash oneself” and physical cleaning alleviates the consequences of immoral behavior and reduces the threat to one's moral self-image (Schnall et al., 2008a,b; Lee and Schwarz, 2011). However, various studies have failed to replicated this effect (e.g., Fayard et al., 2009; Gámez et al., 2011; Earp et al., 2014).

The diversity of findings can be explained if we assume that the Macbeth effect is due solely to the deontological component of guilt and not to the altruistic component. In all the cited studies, in fact, the two components, altruistic and deontological, were not controlled and only a generic feeling of guilt was induced. On the contrary, in one recent study, the inducement of DG but not AG led to the “Lady Macbeth” effect (D'Olimpio and Mancini, 2014)^10^. This study was further replicated by Ottaviani et al. (2018a). They found that the inducement of DG, but not AG, involved not only careful washing, but also the physiological activation typical of disgust observed by way of heart rate variability (HRV). The reduction of HRV, therefore, might be an index that differentiates deontological guilt and altruistic guilt.

It seems plausible to conclude that there exists a privileged relationship between DG and disgust but not between AG and disgust. That there is no relationship, or at least not a an evident and direct relationship, between AG and disgust, is not surprising, since AG is activated by the negative effects on others of one's own actions/omissions and that it activates, in turn, a solicitous disposition toward the victim. Therefore, AG is a strongly other-oriented emotion while disgust activates a motivation to avoid being contaminated and to purify oneself, motivations that do not regard the other, but oneself, and that do not imply, *per se*, concern for the good of others. Furthermore, feeling contaminated and disgusting implies the expectation of being distanced with contempt and aggression (Brandt and Reyna, 2011) and therefore a motivation to avoid others.

What might be the reason for the privileged relationship between DG and disgust?

A first reason might regard moral education, that is, the teaching of respect for moral authority and for its prohibitions and prescriptions, in other words, for moral norms. Disgust is more easily induced than fear, even simple suggestions are sufficient, and it is also more difficult to extinguish (Olatunji et al., 2009). Disgust, therefore, is an excellent instrument for teaching respect for norms. It also has an added advantage over the use of fear as a nurturing tool in that it creates less stress both in the relationship between the nurturer and the nurtured and in the social group within which the nurturing takes place. Furthermore, expressing disgust toward someone is much less taxing than threatening or meting out punishments, and inducing disgust, that is, making a person feel disgusting, implies an involvement of that person's sense of self. Therefore, fewer costs for the educator and also less risk of rebellion on the part of the learner (Ohtsuki et al., 2009; Chapman and Anderson, 2013). Teaching that the transgression of moral norms makes the transgressor disgusting, may be more effective and efficient than recourse to threats, corporal punishment, and penalties.

A second reason may be suggested by the observation that the function of core disgust appears to be to defend against subtle dangers and not, for example, by predatory aggressions^11^. An example is food that appears to be edible but is actually poisonous and whose poisonousness is signaled only by odor or taste. Another example are deformed bodies or bodies affected by dermatological diseases, or by wounds or cadavers. Core disgust seems to protect against subtle and non-evident dangers. Moral disgust may perform an analogous function of inducing repulsion against subtle and dangerous threats posed, not by toxic foods or contagious bodies, but rather by individuals who, in insidious ways, may transgress those deontological norms that help to maintain social order. One finding appears to be coherent with this hypothesis: facial disgust appears to be highest, not only in response to purity violations, but also in response to fairness violations. In contrast, harm violations evoke anger expressions (Cannon et al., 2011). It may, therefore, not be causal that if one is aware of having transgressed deontological norms, beyond feeling a sense of deontological guilt, one also feels disgusting.

The distinction between the two sense of guilt yields implications and indications for future research, which we present in the paragraphs that follow and which regard the relationships between the two senses of guilt and System I and II, a possible explanation of omission bias, some psychopathologies such as obsessive compulsive disorder and major depression disorder, and, finally, a suggestion for research on the role of the two senses of guilt, or better, their scarcity, in individuals with high levels of psychopathy.

## Implications for Moral Psychology

Our distinction brings to mind: (1) the distinction between consequentialist morality and deontological morality (Greene et al., 2009), but for us the difference lies in the goals involved and not in how the information is elaborated; (2) the psychological-evolutionary difference between the morality of *justice* (see Kohlberg, 1981) and the morality of *care* (see Gilligan, 1993), but in our view the two are not incompatible; (3) the psycho-social distinction between the different moral domains of Moral Foundation Theory (Haidt and Joseph, 2004; Graham and Haidt, 2012), but for us the difference is not based on interpersonal and social functions but on goals and beliefs.

Our thesis claims the dignity of both senses of guilt and thus of their relative moralities, altruistic and deontological, and therefore it contests the thesis represented, for example, by Sunstein (2005). “With respect to questions of fact, people use heuristics—mental short-cuts, or rules of thumb, that generally work well, but that also lead to systematic errors. People use moral heuristics too—moral short-cuts, or rules of thumb, that lead to *mistaken and even absurd moral judgments*.” (Sunstein, 2005). As examples of *mistaken and even absurd moral judgments*, Sunstein (2005) suggests “the incest taboo. People have moral revulsion against incest even in circumstances in which the grounds for that taboo seem to be absent; they are subject to ‘moral dumbfounding’ (Haidt et al., 2000), that is, an inability to give an account for a firmly held intuition. It is plausible, at least, to think that System I is driving their judgments, without System II correction.”^12^ A similar thesis is proposed by Greene and others (Greene et al., 2001; Lieberman et al., 2002; Greene, 2009; Hosseinzadeh et al., 2020). Automatic emotional responses to dilemmas are associated with a propensity for deontological options, while controlled cognitive responses are associated with favoring utilitarian options^13^.

From the perspective of our thesis, a purely deontological judgment, such as the condemnation of incest even if it occurs between consenting adults and without negative consequences for anyone, is an error only if it is considered from an altruistic/humanitarian perspective. It is not an error, however, if it is considered from a deontological perspective, which prescribes avoiding what is considered counter to nature or for religious believers, counter to divine will. Furthermore, it is not necessarily the case that deontological judgments are the fruit of System I. Deontological judgments may also be the conclusion reached thanks to System II. An example is the address by Pope Benedict XVI to the Curia (Benedict XVI. 2013, February 6) [General audience]. Paul VI Audience Hall, Vatican, Rome), in which he condemned transgenderism as a transgression of the deontological principle: Do Not Play God. The Pope's argument starts from the premise that human nature is to be man or woman, and, therefore, that gender differences are the expression of the natural order and, finally, of God's will. Transgenderism denies the existence of gender, and therefore aims to subvert human nature, appropriating to itself a right that does not belong to human beings but to God. Therefore, transgenderism is a sin of pride (that is, hubris) and it is grievous because it is an attack on the order willed by God. Obviously, within the confines of this essay, whether the Pope is right or wrong is completely irrelevant. We cite this example to exemplify how a deontological moral judgment, in the same way as a consequentialist judgment, can be justified by reasoned argument. The only difference between the two are the values involved; in one case the well-being and the autonomy of the greatest number of people; in the other, finally, the Do Not Play God, or for non-believers Do Not Tamper with Nature or Do not Tamper with Fate or Tradition, principles that constrain human hubris.

In support of the thesis that deontological judgments are not the fruit of System I, researchers have reported data that do not comport with the dual process model. For example, some researchers have found non-emotional pathways to the deontological option (e.g., Korner and Volk, 2014; Gamez-Djokic and Molden, 2016; see also Robinson et al., 2017), while others have found emotional pathways to the consequentialist option (e.g., Baron, 2011; Moore et al., 2011; Robinson et al., 2015, 2019). Consider two versions of the footbridge dilemma (Cushman and Hauser, 2006). In one, the fat man is made to fall off the bridge by a push, and therefore, by physical contact at the closest possible distance. In the other, the fat man is made to fall by moving a lever that opens a trap door under his feet. In the first case, almost no one throws the fat man under the trolley, differently from what happens in the second case. The two versions differ only in the distance from the fat man, greater in the second than the first. It is intuitive that the smaller the distance, the greater the emotional activation. But the activation of what emotion? We know from a vast literature (e.g., Hoffman, 2000) that physical closeness, especially being able to look the victim in the eye, activates empathy, and thus an emotion associated with altruism, and not with deontological morality. Consider also Bucciarelli et al. (2008): “… the mechanisms underlying emotions and deontic evaluations are independent and operate in parallel, and so some scenarios elicit emotions prior to moral evaluations, some elicit moral evaluations prior to emotions, and some elicit them at the same time. Third, deontic evaluations depend on inferences, either unconscious intuitions or conscious reasoning. …”.

In conclusion, there is no reason to believe that deontological judgments are the product of the so-called System I and that only consequentialist judgments are “rational.” The two moralities differ only in the values that they defend. Whether one morality is better than the other is certainly not up to psychologists to decide.

This conclusion has an implication for future research aimed at analyzing the respective relationships between the two senses of guilt and the decisions based on System I and System II, and particularly, at verifying the thesis that considers moral judgments to be deontological, and therefore deontological guilt, the fruit of an automatic and implicit emotional response.

## Omission Bias

The distinction between the two senses of guilt can also be useful in understanding the nature of the moral asymmetry tied to omission bias, mentioned above.

It is well-established by research that where initial conditions, outcomes, awareness and intentionality are equal, people feel more guilty for an action than for an omission. This is the so-called *omission bias* (Ritov and Baron, 1990). For example, some parents are reluctant to vaccinate their children, even being aware that the risks of vaccination are much lower than those of non-vaccination. In the view of these parents, creating a risk by taking action is considered morally more serious than omitting to act in the face of risk, even if the risk of omission is greater. Action involves, usually, a greater assumption of responsibility tied to the change that the action itself introduces in the direction that fate, divine will, or nature have impressed on events. Action, in fact, involves, more easily than omission, the transgression of a basic deontological principle, *Do Not Play God*,or for non-believers, *Do Not Tamper With Nature* (cf. Sunstein, 2005), that is, of the intuitive principle according to which an individual cannot assume the right to intervene in the life of another person, and more generally, in all that appears to be part of the natural, or for believers, divine order, where that right belongs to God, fate, nature or chance but not to the single individual. The moral difference involved in omission bias, therefore, appears to be related, not so much to the difference between action and omission but rather to transgression of the deontological principle *Do Not Play God or Do Not Tamper With Nature*.

In support of this proposition, imagine being a physician who has to decide whether or not to administer a lethal drug to a terminal patient who, though comatose, displays signs of pain, with the objective of interrupting the patient's useless and painful agony. Now imagine being a physician who, having a patient in the same conditions, must decide whether to interrupt artificial respiration, which would mean the rapid death of the patient, or to leave the patient in useless and painful agony. It is plausible that it would be easier to decide to interrupt the respiration rather than administer the lethal drug (cf. Hauser, 2006). Note that in both cases, to achieve the desired goal, the physician must act, in the first case by giving an injection, in the second by detubing the patient. In both cases, the physician acts, thus modifying the state of things.

In the first case, however, intuition suggests that, by suspending an artificial support system, the physician lets nature take its course. In the second, the injection would impose a new course on events, accelerating them. The difference is not between an action and an omission but between an action that leaves the patient in the hands of fate and an action that instead modifies fate and thus seems to involve the transgression of the principle Do Not Play God/Do Not Tamper With Nature and, consequently, to compromise that which appears intuitively, to the eyes judging the action, as the natural/divine order.

One possible implication for future research could concern the psychological role of the rough and idiosyncratic intuition that there exists a natural order, which is defended by the Do Not Play God/Do Not Tamper With Nature principle. It could be interesting to understand if and how the evaluation of an event is affected by the degree to which it is perceived as congruous or incongruous with one's own intuitive representation of the natural/divine order, and, specifically, if the process of acceptance of adverse events, for example the loss of a loved one, might be facilitated by the assimilation of the event into one's representation of the natural/divine order, or by the accommodation of that representation to the event.

## Implications for Psychopathology

The distinction between AG and Dg seems to have some interesting implications for at least two psychopathological disorders: obsessive compulsive disorder and major depression disorder (MDD). An important tradition (Salkovskis, 1985; Rachman, 1993) holds that fear of guilt is the basis of obsessive symptoms and that obsessive patients are particularly sensitive to guilt feelings (for a review see Mancini, 2018). Some studies suggest that the guilt feared by obsessive patients is prevalently deontological and that obsessive patients are more sensitive to deontology than non-obsessive individuals (for a review see Gangemi and Mancini, 2017). Mancini and Gangemi (2015) found that OC patients were more prone to prevent DG than AG and more prone to prevent DG than both healthy controls and patients with anxiety disorders, by showing a preference for omission rather than action on the switch version of the trolley dilemma. Franklin et al. (2009) investigated moral choices in OC patients using the trolley dilemma, demonstrating that patients' preference for the action choice was inversely related to the severity of their symptoms. Moreover, the stronger patients endorsed responsibility attitudes, the less likely they were to choose to kill one person to save the lives of others.

Two other studies (D'Olimpio and Mancini, 2014; Ottaviani et al., 2018a) found that, in non-clinical SS, DG inducement activated obsessive-like checking and washing behaviors to a greater extent than inducement of AG. Giacomantonio et al. (2019) found that in the condition of uncertainty, non-clinical participants spent more time in checking behaviors when they experienced DG rather than AG.

Some functional magnetic resonance imaging (fMRI) studies (e.g., Rauch et al., 1998; Mataix-Cols et al., 2005) have shown that OC patients undergoing a symptom provocation task have activation in similar areas of the brain (e.g., the anterior cingulate cortex and the insulae) as those activated in healthy individuals experiencing DG (Basile et al., 2011). This overlap suggests that during symptom provocation patients may be experiencing DG. These data are consistent with a fMRI study (Basile et al., 2013) in which the authors investigated the brain responses of OC patients while they were processing DG and AG stimuli. Compared to healthy controls, when processing DG stimuli OC patients showed decreased activation in the anterior cingulate cortex, the insula, and the precuneus. No significant differences were observed between groups when processing AG, angry, or sad stimuli. The authors suggested that this decreased activation may reflect patients' cerebral efficiency, which results from their frequent exposure to DG feelings, known as the neuro efficiency hypothesis (Neubauer and Fink, 2009).

Some studies (Weiss and Sampson, 1986; Weiss, 1993) suggest that AG plays an important role in some cases of MDD. “Enhanced neural response in the depression group, in areas previously linked to altruistic decisions, supports the hypothesis of a possible association between hyper-altruism and depression vulnerability” (Pulcu et al., 2014). MDD is associated with elevated levels of survivor guilt (O'Connor et al., 2000), which persists into remission (Green et al., 2013). O'Connor et al. (2012) concluded their study “suggesting that altruistic concern about others may be an important factor in depression.” O'Connor et al. (2012) also suggested that empathy-based guilt is associated with hyper-altruism in MDD. “Epidemiological studies support this view and suggest that hyper-altruistic tendencies (e.g., making donations exceeding $10/month) constitute a vulnerability factor for the first onset of MDD (Fujiwara, 2009). This suggests that charitable donation, perhaps acting as an index of empathy-based guilt, may represent a trait marker for MDD” (Pulcu et al., 2014).

Fujino et al. (2014) write: “Although speculative, the present result (i.e., MDD patients showed elevated cerebral activation in the left IFG in spite of their reduced pain ratings) might … suggest that MDD patients physiologically showed elevated empathic stress, yet they might not be able to verbalize it due to their multiple cognitive impairments.”

In our view, the distinction between the two senses of guilt could help to resolve some of the contradictions characteristic of research findings on the moral sense of persons with a high level of psychopathy. One well-known thesis claims that a fundamental feature of psychopathy is a lack of empathy (Blair, 1995). However, individuals with psychopathic traits tend to resolve the trolley dilemma by making utilitarian choices in accordance with the principle of maximizing the well-being of the greatest number of people (Bartels and Pizarro, 2011; Luke and Gawronski, 2021)^14^.

It could be interesting to explore the hypothesis that, at least in some individuals with high psychopathic traits, the choice may be determined, rather than by a utilitarian preference, by indifference, or even aversion to deontological morality. As suggested by the conclusion of Cima et al. (2010): “Psychopaths know what is right or wrong, but simply don't care.”

## Conclusions

The guilt feelings that we all experience in our daily lives have two components, altruistic and deontological. These two components, though co-present in most cases, can be activated separately, expressing two senses of guilt, which are different in their psychological determinants, in their relationships with other emotions such as pain and disgust, in their influence on the resolution of moral dilemmas, and in their neural substrates. Our thesis is dualist, while traditionally studies of the sense of guilt are monist. The intrapsychic thesis traces all guilt feelings to the transgression of internalized moral norms; the interpersonal thesis traces them to the compromise of the good of other individuals or groups of individuals. The integrated approach assumes that both components are necessary to feel guilt.

Our thesis is fundamentally cognitivist because it traces guilt feelings to the goals and assumptions of the individual and not, for example, to conflicts between parts of the psychic apparatus or to social and interpersonal functions.

If, on the contrary, we assumed that the psychic apparatus is composed of different parts, then it might be interesting the suggestion of one of the referees of this paper, that is to examine the hypothesis that the sense of deontological guilt is characteristic of one of the parts, for example the super ego, and the other sense of guilt, the altruistic one, is characteristic of another pat, for example the ego.

The thesis suggests a privileged relationship between deontological guilt and disgust, but not between altruistic guilt and disgust. This seems interesting for two reasons. First, it could explain why the “Lady Macbeth” effect is found in some studies but not in others. Indeed, not having taken into account the distinction between the two senses of guilt, it is possible that the altruistic sense of guilt was primarily activated in some experimental groups while the deontological sense of guilt was primarily activated in other groups, giving rise to non-univocal findings. Indeed, as reported above, two studies (D'Olimpio and Mancini, 2014; Ottaviani et al., 2018a) found that the Macbeth effect was present only in those participants in whom a deontological sense of guilt was induced but not in those in whom an altruistic sense of guilt was induced. Second, our thesis suggests that the two senses of guilt may arise from two different evolutionary paths. The altruistic sense of guilt, and more generally altruistic morality, could derive from the care-giving motivation, as suggested, for example, by Tomasello (2016), whereas deontological guilt, and deontological morality, could derive, by way of processes of exaptation, from disgust. Owing to the difficulties of the empirical control of evolutionary theories in the realm of psychology, these considerations are to be taken with caution.

In our view, the distinction between the two senses of guilt has interesting implications in clinical psychology, yielding a more accurate understanding of obsessive compulsive disorder and of at least some forms of depression, and permitting a more precise targeting of the psychotherapeutic intervention (see, for example, Tenore et al., 2020).

With regard to moral psychology, it seems to us that the distinction indicates the need to deepen our knowledge of deontological morality, keeping it separate from the altruistic-humanitarian dimension, especially in the strictly psychological sense rather than in the social-psychological or anthropological sense. In this context, we find particularly interesting the argument made by Fromm (1985) that the fear of being guilty is the fear of having outraged an authority, even an unreal internalized one.

The distinction between the two senses of guilt lends a special dignity to deontological morality and thereby prompts a discussion of the so-called dual process theory, which attributes deontological judgments to the absence of System 2 intervention, which normally should correct the intuitions produced by System 1.

The main limitation of our thesis is that the research on which it is based has been conducted with people belonging to a culture, Italian culture, heavily influenced by Catholicism. Nevertheless, the principals inspiring moral judgments, and therefore guilt feelings, vary not only according to religion (Piazza, 2012) and cultural tradition (Brandt and Rozin, 1997; Shweder et al., 1997) but also according to sex (Gilligan, 1982) social status, and political orientation (Haidt et al., 1993).

It may be useful to guard against a possible semantic misunderstanding. Reading our examples, Anglophone readers might believe that it would be more appropriate to use the term “shame” or “inner shame” rather than “guilt.” We have used the term guilt, sharing as we do the difference in meaning between shame and guilt described and proposed by Miceli and Castelfranchi (2018):“Both shame and guilt are ‘self-critical’ emotions. However, self-criticism may take different self-evaluative forms: on the one hand, people may view themselves as ugly, stupid, handicapped, or morally defective—in a word, *lacking* (in physical attractiveness, intelligence, skills, moral worth, and so on); on the other hand, they may view themselves as wicked, unjust, sinful—that is, endowed *with the power* to violate norms and thwart others' goals, and willing (or inclined) to do so. …. Shame implies perceived *lack of power* to meet the standards of one's ideal self, whereas guilt implies perceived *power and willingness to be harmful*, that is, to violate the standards of one's moral self.” Understood in this sense, the term “guilt” is much closer than “shame” to the meaning of the Italian term “colpa,” while “shame” is much closer to “vergogna.”

Accordingly, the term “colpa” used in the materials of our studies and thus also in the self-reports, refers to a negative self-judgment tied to a “harmful” use of one's own powers and not to a negative judgment tied to a lack of power or to a generic sense of inadequacy. Therefore, in our opinion, “colpa” is more adequately translated as “guilt” rather than “shame.”

To conclude, what are the possible future lines of research based on the distinction we have proposed between the two senses of guilt? We have already suggested three possible areas of research that could be developed from this distinction: (1) the relationship between deontology and System I and II; (2) the role played by the intuitive representation of the natural/divine order, particularly in the process of acceptance of adversity; and (3) the role of deontological guilt, or better, of its absence, in individuals with high levels of psychopathy.

In addition, we have provided some indications for research on questions that are important from the clinical and psychotherapeutic perspective. For example, investigation of different strategies for the management of the two kinds of guilt feelings, that is, what are the actions and elaborations that allow an individual to reduce or resolve the two senses of guilt, and how do these strategies differ? What are the various consequences of the failure of these strategies and thus of the impossibility of freeing oneself from one or the other sense of guilt? Furthermore, is it plausible that the experiences that make people more vulnerable to the deontological sense of guilt are different from those that make people vulnerable to altruistic guilt, but what are they and what differentiates them? What are the therapeutic processes that are effective in reducing, and also inducing, the two senses of guilt and what differentiates them?

## Data Availability Statement

The present paper is not a research paper. However, all the raw data regarding our research can be made available.

## Author's Note

The first and previous Italian, version of the present paper, has been already published on an Italian journal, named Giornale Italiano di Psicologia (Mancini and Gangemi, 2018).

## Author Contributions

Both authors listed have made a substantial, direct and intellectual contribution to the work, and approved it for publication.

## Conflict of Interest

The authors declare that the research was conducted in the absence of any commercial or financial relationships that could be construed as a potential conflict of interest.

## Footnotes

^1^We assume that negative emotions are ascribable to the recognition of a discrepancy between reality, as it is perceived or represented by an individual, and his/her goals, and that such emotions are different depending on the beliefs and goals active in that moment (Bowlby, 1969; Carver and Scheier, 1998; Castelfranchi and Miceli, 2009).

^2^Cf. the aphorism “caring is sharing.”

^3^This ingredient of the sense of guilt may explain the guilt feelings of survivors or the beneficiaries of good fortune.

^4^This episode has been chosen because it has been judged, by many individuals, a good example of a sense of AG devoid of deontological elements (D'Olimpio and Mancini, 2014).

^5^The *Moral Foundation Theory* proposed initially by Haidt and Joseph (2004) and subsequently expanded by Graham and Haidt (2012) identifies five moral domains: *care, fairness, loyalty, authority, sanctity*. The value of these studies and subsequent theorizations lies in their having opened up the study of moral psychology to dimensions that the influence of Anglo-Saxon liberal culture had caused to be ignored or excluded. Moral psychology does not concern only the horizontal dimension, referred to others, but also the vertical dimension, referred to supra-individual values.

^6^A religious norm can be internalized, that is, a believer can have the goal of respecting it and this goal can be terminal for the believer, that is, not instrumental to anything other than respect for God.

^7^In Catholicism, masturbation was considered, and in part still is considered, a sin in that it is a transgression of the commandment “Thou shalt not commit impure acts.” In fact, masturbation was a matter for confession.

^8^We use the expression Do Not Play God interchangeably with the expression Do Not Tamper with Nature or Do Not Tamper with Fate to indicate the intuitive principle that leads us to limit our possibilities of action because we owe obedience to a superior authority that may be abstract or concrete, personal or impersonal. For example, a father is concrete and personal, tradition is concrete and impersonal, destiny is abstract and impersonal, and the divinity may be abstract and personal.

^9^In the *Ultimatum Game* the so-called proposer receives a sum that he must share with the responder. The proposer can decide how much to give to the responder and how much to keep for himself. The responder can decide whether to accept or reject the proposal. If he refuses, neither of them gets anything. Note that, in the *Ultimatum Game*, a proposal may be considered equitable, for example, if it is 50% each, or inequitable, if, for example, the proposer leaves only 20% to the responder, but the proposals are always financially advantageous for the responder because the alternative is to have nothing and, therefore, those who reject the proposal do so exclusively for justice motives. In the so-called three-person version, used in the cited experiment, the decision to accept or reject the proposer's proposal was up to a judge who decided on behalf of the responder, without any benefit to himself. In the experiment, players in which moral pride was induced and those in whom AG was induced considered inequitable and unacceptable proposals that the players did not feel they had the right to sanction by rejecting them, even though they considered them unjust.

^10^The participant listened to a story meant to induce either deontological guilt, altruistic guilt, or no emotional changes. The stories had been chosen through a complex selection process described in D'Olimpio and Mancini (2014): “A large group of students was initially required to write a personal or imagined narrative in which the protagonist feels one of the two target emotions. Each student was given a description of the target emotion in terms of internal dialogue, action propensity, and recurring thought, but the target emotion was not explicitly named. The stories were then submitted to several judges who assess emotions referred to and felt in all of the stories. The stories with the highest average scores on the two emotions (deontological guilt and altruistic guilt) were chosen and modified by adding details that could facilitate identification with the protagonist.”

^11^A thesis similar in part to that advanced by Robinson et al. (2019).

^12^According to Kahneman (2002), System I is intuitive; it is rapid, automatic, and effortless and proposes quick answers to problems of judgment. System II, by contrast, is reflective; it is slower, self-aware, calculative, deductive and operates as a monitor, confirming or overriding those judgments.

^13^The utilitarian option minimizes the harm to other human beings.

^14^For example, in Bartels and Pizarro (2011): “Participants who indicated greater endorsement of utilitarian solutions had higher scores on measures of Psychopathy, machiavellianism, and life meaninglessness.” Similarly, Luke and Gawronski (2021) claim: “Research on moral dilemma judgment suggests that higher levels of psychopathy are associated with a greater preference for utilitarian over deontological judgments.”

## References

[B1] Baron J. (2011). Utilitarian emotions: suggestions from introspection. Emot. Rev. 3, 286–287. 10.1177/1754073911402377

[B2] Bartels D. M. Pizarro D. A. (2011). The mismeasure of morals: antisocial personality traits predict utilitarian responses to moral dilemmas. Cognition 121, 154–161. 10.1016/j.cognition.2011.05.010 21757191

[B3] Basile B. Mancini F. (2011). Eliciting guilty feelings: a preliminary study differentiating deontological and altruistic guilt. Psychology 2, 98–102. 10.4236/psych.2011.22016

[B4] Basile B. Mancini F. Macaluso E. Caltagirone C. Bozzali M. (2013). Abnormal processing of deontological guilt in obsessive-compulsive disorder. Brain Struct. Funct. 4, 1–11. 10.1007/s00429-013-0570-2 23681167

[B5] Basile B. Mancini F. Macaluso E. Caltagirone C. Frackoviack R. Bozzali M. (2011). Deontological and altruistic guilt: evidence for neurobiological different substrates. Hum. Brain Mapp. 32, 229–239. 10.1002/hbm.21009 20842749 PMC6870139

[B6] Baumeister R. F. Stillwell A. M. Heatherton T. F. (1994). Guilt: an interpersonal approach. Psychol. Bull. 115, 243–267. 10.1037/0033-2909.115.2.243 8165271

[B7] Bekoff M. Pierce J. (2010). Wild Justice: The Moral Lives of Animals. Chicago, IL: University of Chicago Press. 10.7208/chicago/9780226041667.001.0001

[B8] Biziou-van-Pol L. Haenen J. Novaro A. Occhipinti-Liberman A. Capraro V. (2015). Does telling white lies signal pro-social preferences? Judgm. Decis. Mak. 10, 538–548.

[B9] Blair R. J. (1995). A cognitive developmental approach to morality: Investigating the psychopath. Cognition 57, 1–29. 10.1016/0010-0277(95)00676-P 7587017

[B10] Bowlby J. (1969). Attachment and Loss, Vol. 1. London UK: Hogart Press.

[B11] Brandt A. M. Reyna C. (2011). The chain of being: a hierarchy of morality. Perspect. Psychol. Sci. 6, 428–446. 10.1177/1745691611414587 26168195

[B12] Brandt A. M. Rozin P. (Eds.). (1997). Morality and Health. Florence, KY: Taylor & Frances/Routledge.

[B13] Bucciarelli M. Khemlani S. Johnson-Laird P. N. (2008). The psychology of moral reasoning. Judgm. Decis. Mak. 3, 121–139.

[B14] Cannon P. R. Schnall S. White M. (2011). Transgressions and expressions: affective facial muscle activity predicts moral judgments. Soc. Psychol. Pers. Sci. 2, 325–331. 10.1177/1948550610390525

[B15] Carnì S. Petrocchi N. Del Miglio C. Mancini F. Couyoumdjian A. (2013). Intrapsychic and interpersonal guilt: a critical review of the recent literature. Cogn. Process. 14, 333–346. 10.1007/s10339-013-0570-4 23732818

[B16] Carver C. S. Scheier M. F. (1998). On the Self-Regulation of Behavior. Cambridge: Cambridge University Press. 10.1017/CBO9781139174794

[B17] Castelfranchi C. (1994). “I sensi di colpa,” in I Sensi di Colpa, eds C. Castelfranchi, R. Conte, and R. D'Amico (Firenze: Giunti), 63–74.

[B18] Castelfranchi C. (2005). Che Figura! Bologna: Il Mulino.

[B19] Castelfranchi C. (2011). “È possibile una mente altruista?,” in Altruismo e Comportamento Prosociale. Temi e Prospettive a Confronto, eds S. Boca and C. Scaffidi Abbate (Milano: Franco Angeli), 85–116.

[B20] Castelfranchi C. Miceli M. (2009). The cognitive-motivational compound of emotional experience. Emot. Rev. 1, 223–231. 10.1177/1754073909103590

[B21] Chapman H. A. Anderson A. K. (2013). Things rank and gross in nature: a review and synthesis of moral disgust. Psychol. Bull. 139, 300–327. 10.1037/a0030964 23458435

[B22] Cima M. Tonnaer F. Hauser M. D. (2010). Psychopaths know right from wrong but don't care. Soc. Cogn. Affect. Neurosci. 5, 59–67. 10.1093/scan/nsp051 20053752 PMC2840845

[B23] Cushman F Young L. Hauser M. (2006). The role of conscious reasoning and intuition in moral judgment. Psychol. Sci. 17, 1082–1089. 10.1111/j.1467-9280.2006.01834.x 17201791

[B24] D'Olimpio F. Mancini F. (2014). Role of Deontological Guilt in obsessive-compulsive disorder-like checking and washing behaviors. Clin. Psychol. Sci. 2, 727–739. 10.1177/2167702614529549

[B25] D'Olimpio F. Mancini F. (2016). ≪Don't Play God!≫: Is inaction preference linked to obsessive compulsive characteristics? Clin. Neuropsychiatry 13, 122–129.

[B26] Doron G. Sar-El D. Mikulincer M. (2012). Threats to moral self-perceptions trigger obsessive compulsive contamination related behavioral tendencies. J. Behav. Ther. Exp. Psychiatry 43, 884–890. 10.1016/j.jbtep.2012.01.002 22306821

[B27] Earp B. D. Everett J. A. C. Madva E. N. Hamlin J. K. (2014). Out, damned spot: can the ≪Macbeth Effect≫ be replicated? Basic Appl. Soc. Psychol. 36, 91–98. 10.1080/01973533.2013.856792

[B28] Fayard J. V. Bassi A. K. Bernstein D. M. Roberts B.W. (2009). Is cleanliness next to godliness? Dispelling old wives' tales: failure to replicate Zhong & Liljenquist (2009). J. Articles Support Null Hypoth. 6, 21–30.

[B29] Fischhoff B. (2003). Hindsight is not equal to foresight: the effect of outcome knowledge on judgment under uncertainty. Qual. Saf. Health Care 12, 304–311. 10.1136/qhc.12.4.304 12897366 PMC1743746

[B30] Foot P. (1967). The problem of abortion and the doctrine of the double effect. Oxford Rev. 5, 5–15.

[B31] Franklin S. A. McNally R. J. Riemann B. C. (2009). Moral reasoning in obsessive–compulsive disorder. Journal of Anxiety Disorders, 23, 575–577. 10.1016/j.janxdis.2008.11.005 19097749

[B32] Fromm E. (1985). Etica y Psychoanalysis. Madrir: Fondo De Cultura Economica.

[B33] Fujino J. Yamasaki N. Miyata J. Kawada R. Sasaki H. Matsukawa N.. (2014). Altered brain response to others' pain in major depressive disorder. J. Affect. Disord. 165, 170–175. 10.1016/j.jad.2014.04.058 24882196

[B34] Fujiwara T. (2009). Is altruistic behavior associated with major depression onset? PLoS ONE 4, 45–57. 10.1371/journal.pone.0004557 19234611 PMC2643004

[B35] Gámez E. Díaz J. M. Marrero H. (2011). The uncertain universality of the Macbeth effect with a Spanish sample. Span. J. Psychol. 14, 156–162. 10.5209/rev_SJOP.2011.v14.n1.13 21568173

[B36] Gamez-Djokic M. Molden D. (2016). Beyond affective influences on deontological moral judgment: the role of motivations for prevention in the moral condemnation of harm. Pers. Soc. Psychol. Bull. 42, 1522–1537. 10.1177/0146167216665094 27655753

[B37] Gangemi A. Mancini F. (2013). “Moral choices: the influence of the do not play God principle,” in Cooperative Minds: Social Interaction and Group Dynamics, Proceedings of the 35th Annual Meeting of the Cognitive Science Society, eds M. Knauff, M. Pauen, N. Sebanz, and I. Wachsmuth (Austin, TX: Cognitive Science Society), 2973–2977.

[B38] Gangemi A. Mancini F. (2017). Obsessive patients and deontological guilt. Psychopathol. Rev. 4, 155–168. 10.5127/pr.045916

[B39] Giacomantonio M. Salvati M. Mancini F. (2019). Am I guilty or not? Deontological guilt, uncertainty, and checking behavior. Appl. Cogn. Psychol. 33, 1279–1287. 10.1002/acp.3600

[B40] Gilligan C. (1982). In a Different Voice: Psychological Theory and Women's Development. Cambridge, MA: Harvard University Press.

[B41] Gilligan C. (1993). In a Different Voice. Cambridge, MA: Harvard University Press.

[B42] Graham J. Haidt J. (2012). “Sacred values and evil adversaries: a moral foundations approach,” in The Social Psychology of Morality: Exploring the Causes of Good and Evil, eds P. Shaver and M. Mikulincer (New York, NY: APA Books), 11–31. 10.1037/13091-001

[B43] Green S. Moll J. Deakin J. F. W. Hulleman J. Zahn R. (2013). Proneness to decreased negative emotions in major depressive disorder when blaming others rather than oneself. Psychopathology 46, 34–44. 10.1159/000338632 22890331

[B44] Greene J. D. (2009). “The cognitive neuroscience of moral judgment”, in The Cognitive Neurosciences, eds M. S. Gazzaniga, E. Bizzi, L. M. Chalupa, S. T. Grafton, T. F. Heatherton, C. Koch, et al. (Cambridge, MA: Massachusetts Institute of Technology), 987–999.

[B45] Greene J. D. Cushman F. A. Stewart L. E. Lowenberg K. Nystrom L. E. Cohen J. D. (2009). Pushing moral buttons: the interaction between personal force and intention in moral judgment. Cognition 111, 364–371. 10.1016/j.cognition.2009.02.001 19375075

[B46] Greene J. D. Sommerville R. B. Nystrom L. E. Darley J. M. Cohen J. D. (2001). An fMRI investigation of emotional engagement in moral judgment. Science 293, 2105–2108. 10.1126/science.1062872 11557895

[B47] Haidt J. Bjorklund F. Murphy S. (2000). Moral Dumbfounding: When Intuition Finds No Reason. Charlottesville: University of Virginia.

[B48] Haidt J. Joseph C. (2004). Intuitive ethics: how innately prepared intuitions generate culturally variable virtues. Daedalus 133, 55–66. 10.1162/0011526042365555

[B49] Haidt J. Koller S. H. Dias M. G. (1993). Affect, culture, and morality, or is it wrong to eat your dog? J. Pers. Soc. Psychol. 65, 613–628. 10.1037/0022-3514.65.4.613 8229648

[B50] Hauser M. (2006). Moral Minds: How Nature Designed Our Universal Sense of Right and Wrong. Ecco/Harper Collins Publishers.

[B51] Hoffman M. L. (1982). “Development of prosocial motivation: empathy and guilt,” in The Development of Prosocial Behavior, ed N. Eisenberg (New York, NY: Academic Press), 218–231. 10.1016/B978-0-12-234980-5.50016-X

[B52] Hoffman M. L. (1998). “Varieties of empathy-based guilt,” in Guilt and Children, ed J. Bybee (San Diego, CA: Academic Press), 91–112. 10.1016/B978-012148610-5/50005-9

[B53] Hoffman M. L. (2000). Empathy and Moral Development: Implications for Caring and Justice. Cambridge: Cambridge University Press. 10.1017/CBO9780511805851

[B54] Hosseinzadeh M. Azhdehakosh E. Valibeygi A. (2020). Moral judgements in obsessive-compulsive disorder: a narrative mini-review. Neurosci. Res. Notes 3, 11–23. 10.31117/neuroscirn.v3i1.39

[B55] Kahneman D. (2002). Maps of Bounded Rationality: A Perspective on Intuitive Judgment and Choice. Nobel Prize Lecture. Available online at: http://nobelprize.org/economics/laureates/2002/kahneman-lecture.html[UH]

[B56] Kelly D. Stich S. Haley K. J. Eng S. J. Fessler D. M. T. (2007). Harm, affect, and the moral/conventional distinction. Mind Lang. 22, 117–131. 10.1111/j.1468-0017.2007.00302.x

[B57] Kohlberg L. (1981). The Philosophy of Moral Development, Moral Stages and the Idea of Justice, Essays on Moral Development. San Francisco, CA: Harper and Row.

[B58] Korner A. Volk S. (2014). Concrete and abstract ways to deontology: cognitive capacity moderates construal level effects on moral judgments. J. Exp. Soc. Psychol. 55, 139–145. 10.1016/j.jesp.2014.07.002

[B59] Kubany E. S. Watson S. B. (2003). Guilt: elaboration of a multidimensional model. Psychol. Rec. 53, 51–90.

[B60] Kugler K. Jones W. H. (1992). On conceptualizing and assessing guilt. J. Pers. Soc. Psychol. 62, 318–327. 10.1037/0022-3514.62.2.318

[B61] Landy J. F. Goodwin G. P. (2015). Does incidental disgust amplify moral disgust? A meta-analytic review of experimental evidence. Perspect. Psychol. Sci. 10, 518–536. 10.1177/1745691615583128 26177951

[B62] Lee S. W. S. Schwarz N. (2011). Clean slate effects: the psychological consequences of physical cleansing. Curr. Direct. Psychol. Sci. 20, 307–311. 10.1177/0963721411422694

[B63] Lieberman M. D. Gaunt R. Gilbert D. T. Trope Y. (2002). Reflexion and reflection: a social cognitive neuroscience approach to attributional inference. Adv. Exp. Soc. Psychol. 34, 199–249. 10.1016/S0065-2601(02)80006-5

[B64] Luke D. M. Gawronski B. (2021). Political ideology and moral dilemma judgments: an analysis using the CNI model. Pers. Soc. Psychol. Bull. 46, 1–12. 10.1177/0146167220987990 33615911

[B65] Mancini F. (1997). Il senso di colpa. Psicoterapia 9, 12–27.

[B66] Mancini F. (2018). The Obsessive Mind: Understanding and Treating Obsessive-Compulsive Disorder. London: Routledge. 10.4324/9780429452956

[B67] Mancini F. Gangemi A. (2015). Deontological guilt and obsessive compulsive disorder. J. Behav. Ther. Exp. Psychiatry 49, 157–163. 10.1016/j.jbtep.2015.05.003 26048080

[B68] Mancini F. Gangemi A. (2018). Senso di colpa deontologico e senso di colpa altruistico: una tesi dualista. Giornale Italiano di Psicologia 45, 493–510.

[B69] Mancini F. Mancini A. (2015). Do not play god: contrasting effects of deontological guilt and pride on decision-making. Front. Psychol. 6:1251. 10.3389/fpsyg.2015.01251 26379584 PMC4548085

[B70] Mataix-Cols D. Rosario-Campos M. C. Leckman J. F. (2005). A multidimensional model of obsessive–compulsive disorder. Am. J. Psychiatry 162, 228–238. 10.1176/appi.ajp.162.2.228 15677583

[B71] Miceli M. Castelfranchi C. (2018). Reconsidering the differences between shame and guilt. Eur. J. Psychol. 14, 710–733. 10.5964/ejop.v14i3.1564 30263080 PMC6143989

[B72] Migliore S. D'Aurizio G. Parisi F. Maffi S. Squitieri B. Curcio G.. (2019). Moral judgment and empathic/deontological guilt. Psychol. Rep. 122, 1395–1411. 10.1177/0033294118787500 30025498

[B73] Milgram S. (1974). Obedience to Authority: An Experimental View. New York, NY: Harper and Row.

[B74] Moll J. de Oliveira-Souza R. Garrido G. J. Bramati I. E. Caparelli-Daquer E. M. Paiva M. L.. (2007). The self as a moral agent: linking the neural bases of social agency and moral sensitivity. Soc. Neurosci. 2, 336–352. 10.1080/17470910701392024 18633822

[B75] Moll J. de Oliveira-Souza R. Moll F. T. Ignacio F. A. Bramati I. E. Caparelli-Daquer E. M.. (2005a). The moral affiliations of disgust: a functional MRI study. Cogn. Behav. Neurol. 18, 68–78. 10.1097/01.wnn.0000152236.46475.a7 15761278

[B76] Moll J. Zahn R. de Oliveira-Souza R. Krueger F. Grafman J. (2005b). Opinion: the neural basis of human moral cognition. Nat. Rev. Neuro Sci. 6:799809. 10.1038/nrn1768 16276356

[B77] Moore A. B. Stevens J. Conway A. R. A. (2011). Individual differences in sensitivity to reward and punishment predict moral judgment. Pers. Ind. Diff. 50, 621–625. 10.1016/j.paid.2010.12.006

[B78] Nelissen R. M. A. Zeelenberg M. (2009). When guilt evokes self-punishment: evidence for the existence of a dobby effect. Emotion 9, 118–122. 10.1037/a0014540 19186924

[B79] Neubauer A. C. Fink A. (2009). Intelligence and neural efficiency. Neurosci. Biobehav. Rev. 33, 1004–1023. 10.1016/j.neubiorev.2009.04.001 19580915

[B80] Nichols S. (2004). Sentimental Rules: On the Natural Foundations of Moral Judgement. Oxford: Oxford University Press. 10.1093/0195169344.001.0001

[B81] O'Connor L. E. Berry J. W. Lewis T. B. Stiver D. J. (2012). “Empathy-based pathogenic guilt, pathological altruism, and psychopathology,” in Pathological Altruism, eds B. Oakley, A. Knafo, G. Madhavan, and D. S. Wilson (New York, NY: Oxford University Press), 10–30. 10.1093/acprof:oso/9780199738571.003.0024

[B82] O'Connor L. E. Berry J. W. Weiss J. Schweitzer D. Sevier M. (2000). Survivor guilt, submissive behaviour and evolutionary theory: The down-side of winning in social comparison. Br. J. Med. Psychol. 73, 519–530. 10.1348/000711200160705 11140792

[B83] Ohtsuki H. Iwasa Y. Nowak M. A. (2009). Indirect reciprocity provides only a narrow margin of efficiency for costly punishment. Nature 457, 79–82. 10.1038/nature07601 19122640 PMC2614697

[B84] Olatunji B. O. Wolitzky-Taylor K. B. Willems J. Lohr J. M. Armstrong T. (2009). Differential habituation of fear and disgust during repeated exposure to threat-relevant stimuli in contamination-based OCD: an analogue study. J. Anxiety Disord. 23, 118–123. 10.1016/j.janxdis.2008.04.006 18541403

[B85] Ottaviani C. Collazzoni A. D'Olimpio F. Moretta T. Mancini F. (2018a). I obsessively clean because DG makes me feel physiologically disgusted! J. Obsess. Compuls. Relat. Disord. 20, 21–29, 10.1016/j.jocrd.2018.01.004

[B86] Ottaviani C. Mancini F. Petrocchi N. Medea B. Couyoumdjian A. (2013). Autonomic correlates of physical and moral disgust. Int. J. Psychophysiol. 89, 57–62. 10.1016/j.ijpsycho.2013.05.003 23684734

[B87] Ottaviani C. Mancini F. Provenzano S. Collazzoni A. D'Olimpio F. (2018b). Deontological morality can be experimentally enhanced by increasing disgust: a transcranial direct current stimulation study. Neuropsychologia 119, 474–481. 10.1016/j.neuropsychologia.2018.09.009 30244001

[B88] Parisi D. (1977). Affetto. Giornale Italiano Psicol. 5, 127–142.

[B89] Piazza J. (2012). “If you love me keep my commandments”: religiosity increases preference for rule-based moral arguments. Int. J. Psychol. Relig. 22, 285–302. 10.1080/10508619.2011.638598

[B90] Prinz J. Nichols S. (2010). “Moral emotions,” in Moral Psychology Handbook, ed J. Doris (Oxford: Oxford University Press), 111–148. 10.1093/acprof:oso/9780199582143.003.0005

[B91] Pulcu E. Lythe K. Elliott R. Green S. Moll J. Deakin J. F. W.. (2014). Increased amygdala response to shame in remitted major depressive disorder. PLoS ONE 9:e86900. 10.1371/journal.pone.0086900 24497992 PMC3907379

[B92] Rachman S. (1993). Obsessions, responsibility and guilt. Behav. Res. Ther. 31, 149–154. 10.1016/0005-7967(93)90066-4 8442740

[B93] Rauch S. L. Dougherty D. D. Shin L. M. Alpert N. M. Manzo P. Leahy L.. (1998). Neural correlates of factor-analyzed OCD symptom dimensions: a PET study. CNS Spect. 3, 37–43. 10.1017/S1092852900006167

[B94] Ritov I. Baron J. (1990). Reluctance to vaccinate: omission bias and ambiguity. J. Behav. Decis. Making 3, 263–277. 10.1002/bdm.3960030404

[B95] Robinson J. S. Joel S. Plaks J. E. (2015). Empathy for the group versus indifference toward the victim: effects of anxious avoidant attachment on moral judgment. J. Pers. Soc. Psychol. 56, 139–152. 10.1016/j.jesp.2014.09.017

[B96] Robinson J. S. Page-Gould E. Plaks J. E. (2017). I appreciate your effort: asymmetric effects of actors' exertion on observers' consequentialist versus deontological judgments. J. Exp. Soc. Psychol. 73, 50–64. 10.1016/j.jesp.2017.06.005

[B97] Robinson J. S. Xu X. Plaks J. E. (2019). Disgust and deontology: trait sensitivity to contamination promotes a preference for order, hierarchy, and rule-based moral judgment. Soc. Psychol. Pers. Sci. 10, 3–14. 10.1177/1948550617732609

[B98] Rozin P. Haidt J. McCauley C. R. (2000). “Disgust,” in Handbook of Emotions, 2nd Edn, eds M. Lewis and J. Haviland- Jones (New York, NY: Guilford Press), 673–653.

[B99] Salkovskis P. M. (1985). Obsessional–compulsive problems: a cognitive–behavioural analysis. Behav. Res. Ther. 28, 571–588. 10.1016/0005-7967(85)90105-6 4051930

[B100] Salvo G. Provenzano S. Di Bello M. D'Olimpio F. Ottaviani C. Mancini F. (2021). Filthiness of Immorality: Manipulating Disgust and Moral Rigidity Through Non-Invasive Brain Stimulation as a Promising Therapeutic Tool for OCD Patients. Clinical Psychological Science.

[B101] Schnall S. Benton J. Harvey S. (2008a). With a clean conscience cleanliness reduces the severity of moral judgments. Psychol. Sci. 19, 1219–1222. 10.1111/j.1467-9280.2008.02227.x 19121126

[B102] Schnall S. Haidt J. Clore G. L. Jordan A. H. (2008b). Disgust as embodied moral judgment. Pers. Soc. Psychol. Bull. 34, 1096–1109. 10.1177/0146167208317771 18505801 PMC2562923

[B103] Shallice T. (2001). Theory of mind and the prefrontal cortex. Brain 124, 247–248. 10.1093/brain/124.2.247 11157553

[B104] Shweder R. A. Much N. C. Mahapatra M. Park L. (1997). “The “big three” of morality (autonomy, community, divinity) and the “big three” explanations of suffering,” in Morality and Health, eds A. M. Brandt and P. Rozin (New York, NY: Taylor & Frances/Routledge), 119–169.

[B105] Stich S. P. Doris J. M. Roedder E. (2010). “Altruism,” in The Moral Psychology Handbook, eds J. M. Doris and The Moral Psychology Research Group (Oxford: Oxford University Press), 147–205. 10.1093/acprof:oso/9780199582143.003.0006

[B106] Styron W. (1979). Sophie's Choice. New York, NY: Penguin Random House.

[B107] Sunstein C. R. (2005). Moral heuristics. Behav. Brain Sci. 28, 531–573. 10.1017/S0140525X05000099 16209802

[B108] Tangney J. P. Dearing R. (2002). Shame and Guilt. New York, NY: Guilford.

[B109] Tenore K. Basile B. Cosentino T. De Sanctis B. Fadda S. Femia G.. (2020). Imagery rescripting on guilt-inducing memories in OCD: a single case series study. Front. Psychiatry 11:543806. 10.3389/fpsyt.2020.543806 33192658 PMC7554624

[B110] Tobia K. P. (2014). The effects of cleanliness and disgust on moral judgment. Philos. Psychol. 28, 556–568. 10.1080/09515089.2013.877386

[B111] Tomasello M. (2016). A Natural History of Human Morality. Cambridge, MA: Harvard University Press. 10.4159/9780674915855

[B112] Turiel E. Nucci L. (1978). Social interactions and the development of social concepts in preschool children. Child Dev. 49, 400–407. 10.2307/1128704

[B113] Warneken F. Tomasello M. (2009). The roots of human altruism. Br. J. Psychol. 100, 455–471. 10.1348/000712608X379061 19063815

[B114] Weiss J. (1993). How Psychotherapy Works: Process and Technique. New York, NY: Guilford Press.

[B115] Weiss J. Sampson H. (1986). The Psychoanalytic Process: Theory, Clinical Observation and Empirical Research. New York, NY: Guilford Press.

[B116] Zhong C. B. Liljenquist K. (2006). Washing away your sins: Threatened morality and physical cleansing. Science 313, 1451–1452. 10.1126/science.1130726 16960010

